# Accuracy and Reliability of Artificial Intelligence in Surgical Decision-Making: A Literature Review

**DOI:** 10.7759/cureus.95337

**Published:** 2025-10-24

**Authors:** Nicolás Idárraga Ruiz, Israel Cardona Salazar, Lincoln Xavier Naranjo Palacio, Carolina Agudelo Agudelo, Alfonso Miguel Ledesma Parra, Julio Cesar Flores Rodriguez

**Affiliations:** 1 Surgery, University of Manizales, Manizales, COL; 2 Surgery, Instituto Mexicano del Seguro Social, Sonora, MEX; 3 General Practice/Emergency, Clínica Internacional de Traumatología, Quito, ECU; 4 Emergency, Clínica Las Américas Auna, Medellín, COL; 5 Surgery, Hospital de Especialidades del Centro Médico Nacional La Raza, Instituto Mexicano del Seguro Social (IMSS), Ciudad de México, MEX; 6 Aesthetic and Regenerative Medicine, Clínica Aura, San Pedro Garza García, MEX

**Keywords:** artificial intelligence in surgery, decision-making, intraoperative, reliability, surgery

## Abstract

This narrative literature review synthesized evidence to address gaps in knowledge regarding AI performance and its integration into surgical operations. The purpose of the review was to assess AI accuracy and reliability, benchmark real-time guidance technologies, identify data and ethical issues, compare model performance across different specialties, and review the role of AI in improving surgical accuracy and safety. It reviewed 28 studies conducted across various geographic and disciplinary contexts and discussed machine learning (ML) and deep learning (DL) as applied to major surgeries. Results show that AI models' overall performance is substantial in intraoperative (IOP) decision-making, with five of six studies reporting AUC values of 0.85-0.95, indicating significant discriminatory power. Moreover, the accuracy performance metric across 22 studies showed high predictive performance of AI models in surgical settings, with accuracies ranging from 80% to 99%, except for one study, which reported an accuracy below 70%. These findings emphasized the practical feasibility of AI in IOP decision-making. Hence, AI's role in IOP is promising, assisting surgeons' decision-making in the operating room. Therefore, ML and DL are highly precise in anatomic detection, surgical-phase detection, complication prediction, and real-time event detection. Developments in DL algorithms, such as convolutional neural networks and generative adversarial networks, have enabled more accurate surgical guidance and the prediction of IOP events, thereby increasing surgical accuracy and potentially reducing errors. However, the model's performance needs to be validated through long-term computational and real-time clinical study designs, ensuring appropriate strategies for data validation and model performance assessment. The narrative review study design focused solely on the narrative synthesis, rather than on data validation (internal or external) or quality assessment of the included studies. Therefore, future researchers should conduct a systematic review to validate the findings. The readers must be cautious when interpreting the findings. Hence, AI use in surgery training and workflow optimization has the potential to improve surgical performance and patient outcomes, but scalability and long-term outcomes have yet to be demonstrated. Although AI technologies can improve the accuracy and reliability of decisions made in IOP settings, it is critical to address methodological, infrastructural, and ethical constraints to enable safe and effective clinical application in major surgeries.

## Introduction and background

Surgery accounts for a large share of global morbidity and mortality, with low- and middle-income countries (LMICs) suffering the most significant burden due to inadequate access to timely and quality surgery [1]. Artificial Intelligence (AI) has significant applications in surgery, specifically in improving intraoperative (IOP) decision-making, but validation quality, data standardization, and public data availability remain suboptimal. Currently, AI applications are mainly focused on preoperative risk assessment and are suggested to improve decision-making [2,3]. The exploration of AI in IOP decision-making systems during large-scale surgery is a debatable topic.

Over the last few years, the nature of research and practice in surgery has shifted from preoperative risk assessment to real-time IOP decision-making enabled by machine learning (ML) and deep learning (DL) [4,5]. During surgery, AI uses computational systems and algorithms to simulate human cognitive functions, e.g., decision-making, learning, and problem-solving. ML algorithms support predictive analysis, while DL assists with interpreting image and video data and with reinforcement learning for real-time decision-making in autonomous surgical settings [5]. AI assists in real-time anatomical recognition, hazard detection, surgical phase classification, and predictive analysis in surgical decision-making. These algorithms help improve surgeons' decision accuracy, reduce errors, and improve patient outcomes by providing data-driven recommendations during surgery [6]. The use of AI would be beneficial, as IOP complications are quite high, and decision-making under dynamic conditions for surgeons in this field is impossible without complex tools that assist them [6,7]. Since surgical procedures, by definition, represent a significant source of morbidity and mortality worldwide, AI-driven approaches to resource management and patient health will be streamlined [8,9].

Although there is increased interest, there are critical issues with the use of AI in the IOP environment. The existing body of knowledge identifies a gap in understanding the accuracy and reliability of ML and DL models in surgery, particularly for real-time decision support [10,11]. Some of these studies demonstrate promising predictive performance and autonomous capability, but others highlight limitations, such as poor data standardization, insufficient external validation, and ethical concerns [12,13]. Concerns about the safety of AI augmentation or the replacement of human judgment during an operation persist, as does debate about the risks and opportunities of overreliance and interpretation issues on both sides of the technology [14]. These literature gaps included delays in clinical adoption and missed opportunities to improve surgery-related outcomes [15].

The review provides a contextualization of IOP AI, including providing ML algorithms, DL models, and utility to surgical processes [16]. ML is the hallmark of predictive analytics applied to historical data, and DL is the hallmark of smart pattern recognition applied to streams of image and video data [17]. That is, while no single technology can address all three dimensions of improving IOP decision-making, these technologies work together because each contributes to the three heuristic dimensions: real-time anatomic recognition, hazard visualization, and autonomous robotic assistance [18]. A template is provided to systematically evaluate the validity and reliability of AI in surgery.

The purpose of this literature review was to critically assess the current status of ML and DL technology with respect to the accuracy, reliability, and applicability for clinical practice in IOP decision-making associated with major surgery. Hopefully, the knowledge gaps covered above have been addressed, and this review can also serve as a guide for future research on the responsible use of AI in surgery and, by extension, through surgery, to benefit patient care [19].

Objective

The paper synthesized evidence on AI (ML and DL) uses to identify technological advances, assess their clinical effectiveness, and identify challenges to be overcome to achieve safe and effective application of AI in major surgical operations.

## Review

Methodology

**Figure 1:**
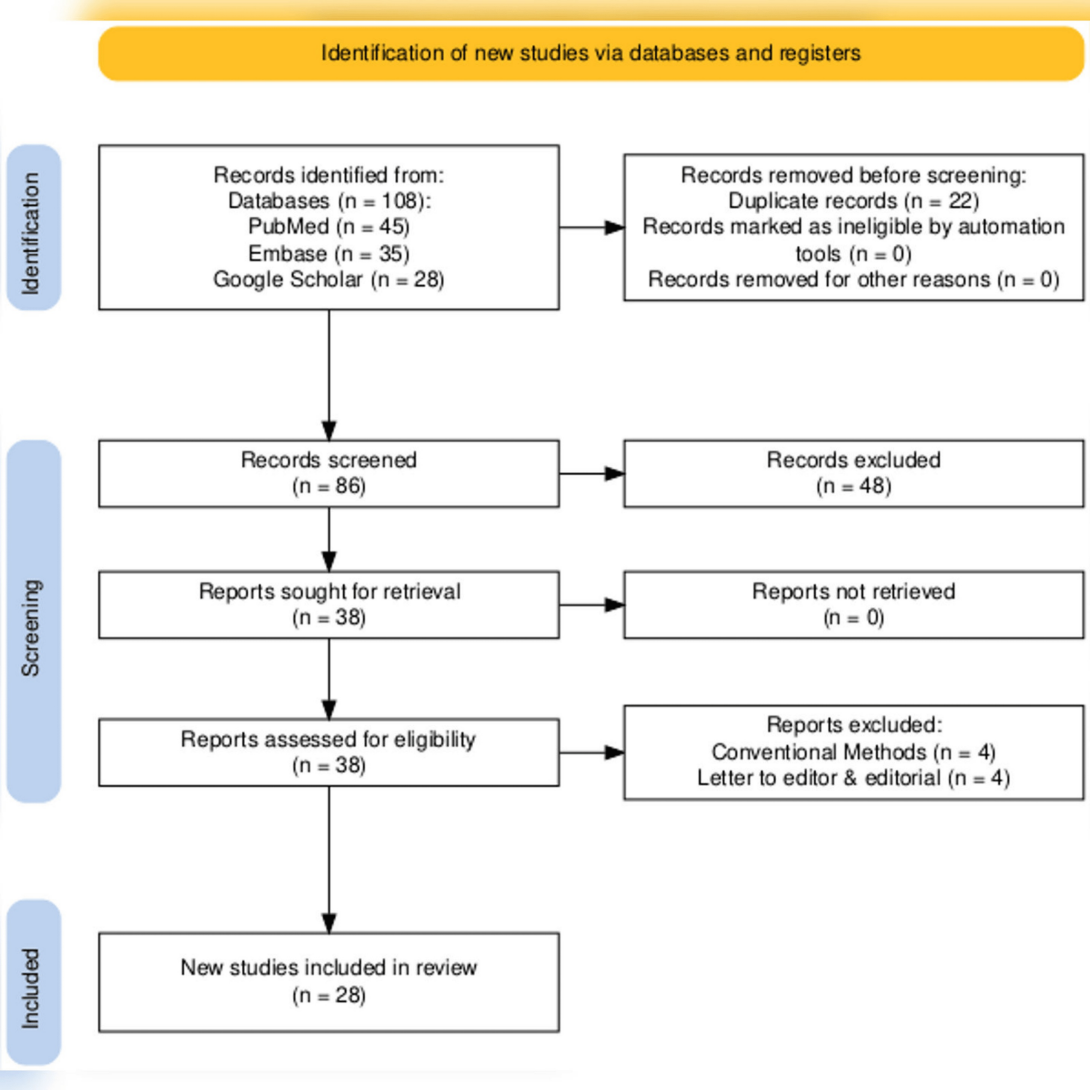
PRISMA flow chart showing the study selection process PRISMA: Preferred Reporting Items for Systematic Reviews and Meta-Analyses

The literature review was conducted using relevant topic-specific keywords such as “artificial intelligence”, “machine learning”, “deep learning”, “surgery”, “intra-operative”, “decision making”, “accuracy”, “reliability”, “low resource setting”, and “implication of AI”. The Boolean operators were used to incorporate keywords and search across PubMed, Embase, and Google Scholar, focusing on articles published from January 2015 to August 2025. All original articles, including RCTs, cross-sectional, cohort, and longitudinal studies, relevant to the keywords, scope, and objectives of the studies were included. However, editorials, letters to the editor, abstracts, and conference papers were excluded before synthesizing evidence. The literature search was limited to peer-reviewed English-language literature. The evidence was synthesized solely using a narrative approach. The selection of the study process was demonstrated in Figure 1.

Results and discussion

Upon synthesizing the evidence, the following studies were analyzed to determine the role of AI in IOP decision-making (Table 1).

**Table 1:** Evaluation of the literature to assess the performance metrics and applicability of AI predictive models AI: artificial intelligence, AUC: area under the curve, F1 score: a measure of a model's accuracy, ML: machine learning, DL: deep learning, CNN: convolutional neural network, EMR: electronic medical records, EHR: electronic health records, RSI: retained surgical items, SSI: surgical site infection, YOLOv5: You Only Look Once Version 5 (a DL object detection model), IOP: intraoperative

Study	Accuracy metrics	Reliability and robustness	Clinical integration feasibility	Ethical and regulatory compliance	Impact on surgical outcomes
Hossain et al. (2024) [5]	Moderate (63.2%) to high accuracy (93.4%) in anatomy identification and phase classification	Challenges due to data quality and standardization	Requires substantial data and infrastructure; ethical concerns noted	Ethical and patient acceptance issues highlighted	Improved IOP guidance and complication prediction (p < 0.05)
Cheruvu et al. (2024) [20]	IOP AI guidance accuracy of up to 95% reported	Limited clinical validation; mostly retrospective data	Early-stage integration; regulatory and ethical challenges remain	Emphasizes the need for ethical frameworks	Potential to transform surgical phases and outcomes
Shetti et al. (2024) [4]	Demonstrated enhanced surgical precision and error reduction on review of case studies	Consistent improvements across case studies	Integration feasible with current surgical workflows	Ethical and future trend considerations discussed	Significant improvements in patient outcomes
Knudsen et al. (2024) [18]	High accuracy in robotic surgery metrics (60-95% accuracy, AUC = 0.88) and automation	Robustness shown in ex vivo and in silico models	Infrastructure-intensive; early clinical adoption	Ethical dilemmas in autonomy addressed	Enhanced surgical education and IOP feedback
Madani et al. (2022) [6]	F1 scores up to 0.83 for zone identification in laparoscopic surgery	Validated across diverse international datasets	Real-time application feasible with video processing	Data privacy and annotation ethics considered	Reduced risk of adverse events intraoperatively
Loftus et al. (2023) [10]	Variable accuracy; some models with AUC < 0.83	Mostly internal validation; limited external and real-time validation	Clinical implementation frameworks proposed but untested	Lack of equity and demographic performance reporting	Limited evidence on direct outcome improvements
Andras et al. (2020) [21]	Encouraging accuracy in robotic surgery skill feedback and guidance (93% for ML vs. 72% for the clinical approach)	Consistent performance in skill acquisition and process efficiency	Integration with robotic platforms feasible	Ethical and regulatory frameworks under development	Improved surgical training and precision
Taher et al. (2022) [12]	Identified technical and clinical challenges limiting DL accuracy to 80%	Reliability affected by data scarcity and complexity	Infrastructure and surgeon education critical	Ethical and business challenges noted	Potential hindered by current limitations
Celotto et al. (2024) [8]	Superior predictive power for anastomotic leak prevention (76.7-91.9% accuracy)	Robustness in clinical datasets for risk factors	Feasible IOP feedback integration	Ethical use in patient safety emphasized	Reduced complication rates and improved decision-making
Rodler et al. (2024) [22]	Emerging generative AI shows promising accuracy in data synthesis	Reliability depends on task-specific training	Real-time feedback and documentation feasible	Ethical considerations in data use highlighted	Enhances IOP decision support and documentation
Othman and Kaleem (2024) [11]	Moderate accuracy (75.7-82%) in IOP guidance and training	Limited data availability affects robustness	Early-stage clinical integration; validation tools lacking	Ethical concerns and data limitations significant	Potential to enhance surgical training and autonomy
Checcucci et al. (2023) [23]	Over 90% accuracy in bleeding event prediction	Reliable performance comparable to human assessment	Real-time IOP application feasible	Ethical use in patient safety emphasized	Improved bleeding management and surgical safety
Rus et al. (2023) [24]	High accuracy (90.63%) in real-time hemorrhage detection using YOLOv5	Robust detection with low false positives	Real-time AR integration feasible; hardware limits noted	Ethical use and surgeon interaction considered	Enhanced hazard detection and patient safety
Mascagni et al. (2024) [7]	The feasibility of real-time AI assistance demonstrated a mean accuracy of 71.4%	Early-stage validation with multidisciplinary input	Technical and cultural barriers identified	Ethical and regulatory challenges noted	Potential to improve IOP assistance
Celotto et al. (2025) [19]	High accuracy (80-94% with an F1 score of 0.90 ± 0.11) in IOP guidance and complication prediction	Robust across colorectal surgery datasets	Integration feasible with imaging and EHR systems	Ethical and regulatory challenges discussed	Improved surgical precision and postoperative outcomes
Zarghami (2024) [14]	High accuracy (> 90%) 90%) in imaging and physiological monitoring	Robustness challenged by data quality and interpretability	Integration requires infrastructure and clinician engagement	Ethical, privacy, and regulatory challenges significant	Improved IOP decision-making and personalized care
Kuemmerli et al. (2023) [25]	Promising accuracy (71-94%) in pancreatic surgery AI applications	Robustness limited by the evidence level	Integration feasible in pre-, intra-, and postoperative phases	Ethical and regulatory challenges noted	Improved diagnosis, decision support, and risk stratification
Demir et al. (2023) [17]	High accuracy in (82-85%) surgical phase and step recognition	Robust temporal modeling with DL	Integration with surgical workflow analysis feasible	Ethical concerns less emphasized	Enhanced workflow recognition and surgical assistance
Mehta et al. (2024) [26]	Variable accuracy (70-85%) in perioperative ML interventions	Reliability depends on the intervention type and data	Clinical integration in perioperative care feasible	Ethical and implementation challenges noted	Improved perioperative outcomes in some settings
Morris et al. (2023) [16]	Broad AI applications with promising accuracy (>80%)	Robustness varies with application and data	Integration feasible with training and decision support	Ethical and interpretability challenges discussed	Enhanced surgical training and decision-making
Henn et al. (2022) [27]	ML reported 97.8%, outperforms conventional decision-making in abdominal surgery	Robustness limited by data heterogeneity	Integration feasible with EHR and clinical workflows	Ethical and interpretability challenges noted	Enhanced clinical decision-making and risk assessment
Ladinez et al. (2024) [28]	ML algorithms (78-96%, AUC of 0.9) outperform conventional methods in complication prediction	Robustness varies with dataset and algorithm	Integration feasible in postoperative care	Ethical and interpretability challenges noted	Enhanced postoperative complication prediction
Abo-Zahhad et al. (2024) [29]	High accuracy in (99.9% with AUC 0.81–0.85) RSI detection and prevention	Robustness enhanced by large datasets	Integration feasible with real-time monitoring	Ethical and data privacy challenges noted	Reduced RSI and improved safety
Spence et al. (2023) [30]	Neural networks (89.4% accuracy) outperform industry standards in surgery duration prediction	Robustness demonstrated in multiple studies	Integration feasible with scheduling systems	Ethical concerns minimal	Improved operating room efficiency (p < 0.05)
Wu et al. (2024) [31]	Significant improvement in surgical performance with AI coaching (accuracy 11% to 78%)	Robustness shown in randomized controlled trial	Integration feasible in surgical education	Ethical concerns minimal	Enhanced surgical safety and training outcomes (p = 0.021)
Chen et al. (2020) [32]	CNN and self-attention models achieve AUC ~0.88 in SSI risk	Robust internal and external validation	Integration feasible with EMR data	Ethical and privacy considerations addressed	Improved SSI risk prediction
Tanzi et al. (2020) [33]	Encouraging results in DL (> 85% accuracy) for IOP management	Robustness across surgical subfields	Integration feasible in intelligent operating rooms	Ethical and workflow challenges noted	Improved surgical workflow and context detection
Ahmad (2023) [34]	ML shows a significant edge (> 75% accuracy) over clinical diagnosis in neurosurgery	Robustness limited by study design variability	Integration feasible with radiology workflows	Ethical and variability challenges noted	Enhanced diagnosis and treatment planning (p < 0.05)

Performance Metrics

**Figure 2:**
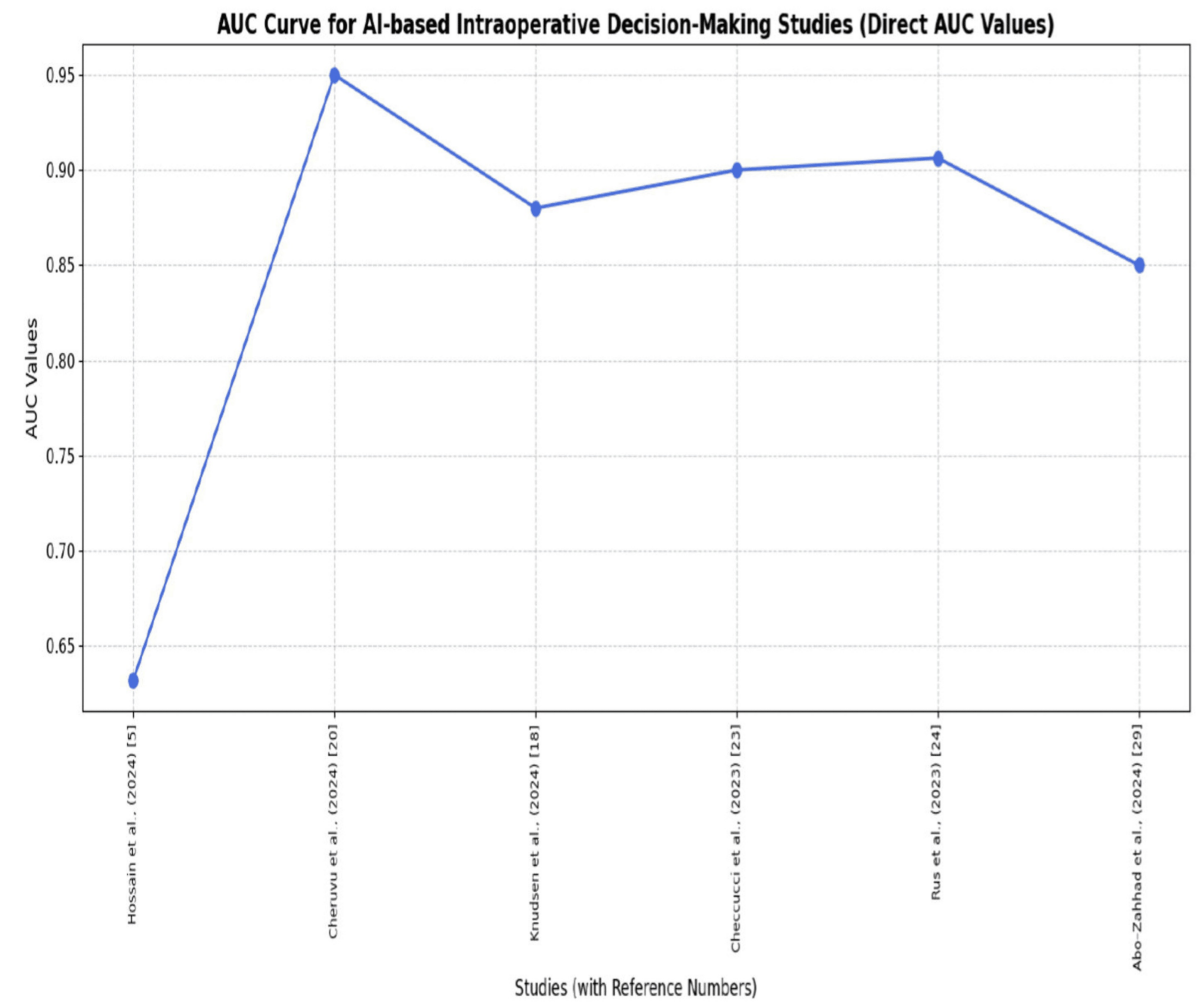
AUC for AI-based IOP decision-making These six studies provided AUC values directly; therefore, only these are used to draw an AUC curve diagram. The reasoning is that AUC is ideal for understanding overall model performance and generalization. AUC is better suited for evaluating model robustness and general performance in clinical practice. These findings are interpreted from a narrative review. Readers should be cautious when interpreting the exact AI model performance due to the study's design. However, the purpose of this graph is to illustrate overall general performance. AUC: area under the curve, AI: artificial intelligence, IOP: intraoperative

**Figure 3:**
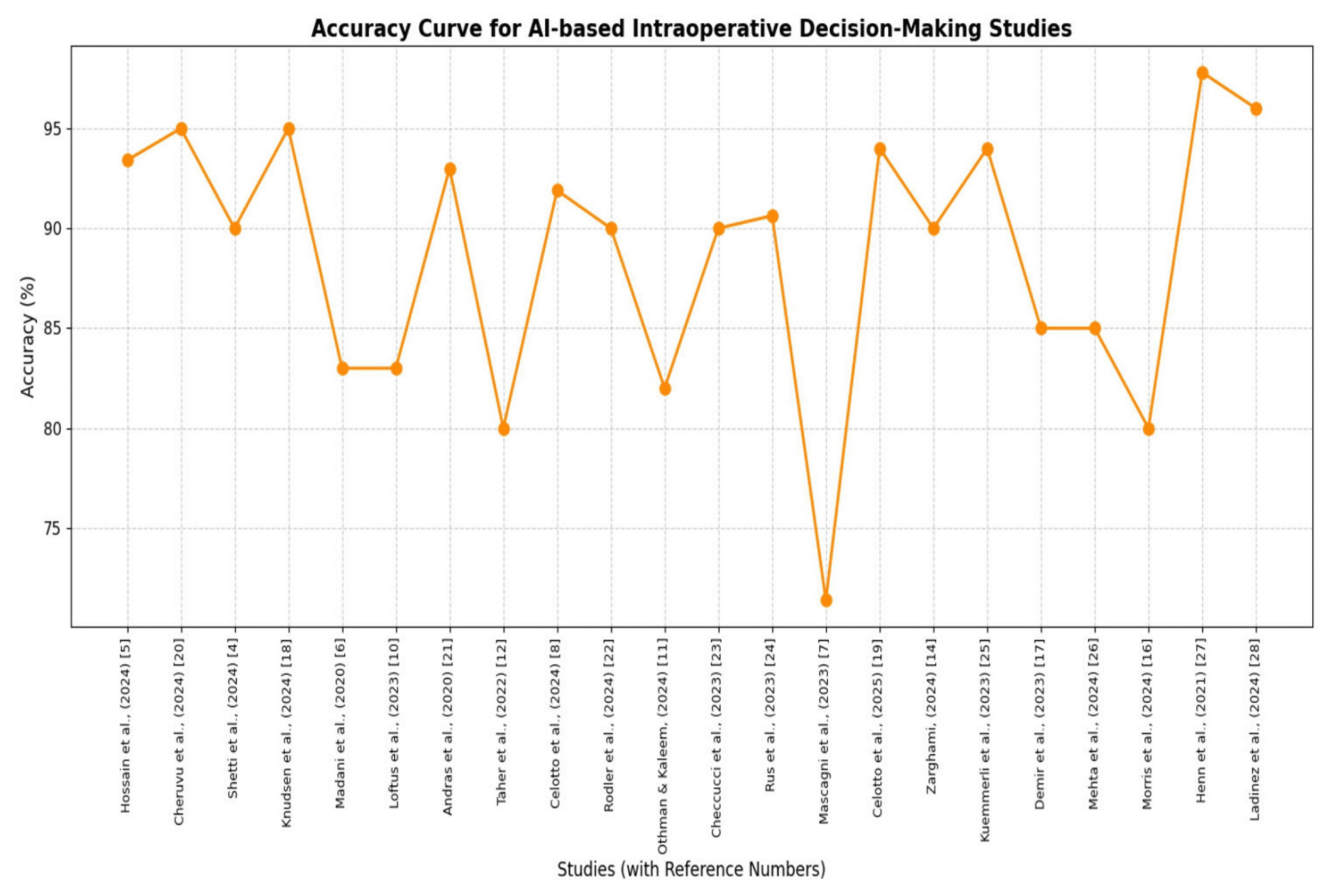
Accuracy curve diagram illustrating the predictability of AI models for IOP decision-making The accuracy metrics from 22 studies were selected to demonstrate the AI model's predictive performance. These studies did not provide a direct computation of the AUC. The reader must be cautious when interpreting the AI model's accuracy, given the narrative review study design. Readers must consider the quality of the studies when evaluating the accuracy of the performance metric. Overall, the graph illustrates the comprehensive practical feasibility of using AI in IOP decision-making. AUC: area under the curve, AI: artificial intelligence, IOP: intraoperative

AI can be used to perform high-to-middle and high-to-very-high accuracy IOP tasks, with F1 scores of 0.83 and AUCs over 0.90 [6,32]. Other studies in this category have reported that AI was highly predictive compared with conventional approaches, particularly for predicting complications and surgical plans [8,28]. Specific initial or preliminary reports of variable accuracy have been noted in some of these early-stage or experimental studies, and thus further validation is required [10,35]. The overall performance of AI models is substantial in IOP decision-making, with five of six studies reporting AUC values of 0.85-0.95, indicating significant discriminatory power (Figure 2). Moreover, the accuracy performance metric among 22 studies showed high predictability, with accuracy ranging from 80% to 99%, except for one study below 70%, emphasizing the practical feasibility of AI in IOP decision-making. Hence, AI's role in IOP is promising for assisting surgeons' decision-making in the operating room (Figure 3).

Reliability and Robustness

The researchers reported similar AI performance across various surgical cases and datasets, even though data quality, heterogeneity, and external validation are usually poor predictors of performance [5,27]. Only animal and in silico models have shown promising robustness in autonomous and robotic surgery applications, although clinical robustness is yet to be demonstrated [35]. The importance of large, well-labeled data to facilitate model stability and generalization has been noted in multiple reviews [12,29].

Clinical Integration Feasibility

Research also suggested that AI can be integrated with existing surgical processes, particularly robotic surgery, IOP guidance, and perioperative risk assessment [18,19,31]. Some of these systems were also shown to be capable of processing in real time, but the facilities and computing power requirements are still significant obstacles [24]. The user must accept and train with the technology, and there must be evidence of positive feedback from the surgeon during use [7,23].

Impact on Surgical Outcomes

The results of the study showed that strong AI ability predicts the quality of IOP judgment and complication prevention, as well as patient and evidence safety and protection [4,19,31]. The error rate in acquiring surgical skills and ensuring surgical safety improved during training under AI coaching [31]. Given its initial research, only a limited number of studies (Table 2) have shown concrete improvements in results, and this is why further clinical confirmation is required [7,10].

**Table 2:** Convergence and divergence across studies AI: artificial intelligence, ML: machine learning, DL: deep learning, CNN: convolutional neural network

Comparison criterion	Studies in convergence	Studies in divergence	Potential explanations
Accuracy metrics	Many studies report high accuracy, sensitivity, and specificity of AI models in IOP tasks, such as real-time anatomy identification and complication prediction (e.g., 90%+ accuracy in bleeding detection, 89-95% sensitivity in guidance systems [6,23,32]	Some studies highlight moderate or variable accuracy and emphasize challenges in achieving consistent, reliable metrics across diverse surgical phases and settings [5,10]	Differences stem from AI model types (ML vs. DL), surgical specialties, dataset size and quality, and the stage of development (experimental vs. clinical). Also, some focus on early-phase validation, while others report mature system performance
Reliability and robustness	Consensus exists that AI tools show promising robustness in controlled or experimental settings, such as ex vivo and animal models for robotic surgery and IOP prediction [18,23]	Divergence arises in real-world reliability; many systems lack external validation, real-time clinical testing, and show risk of overfitting or less robustness in heterogeneous clinical environments [10,11]	Variability is attributed to limited external validation, small or homogeneous datasets, and a lack of large-scale clinical trials. Differences in infrastructure support and surgical workflow integration also affect robustness
Clinical integration feasibility	Agreement that AI has potential for IOP decision support and workflow improvement, with some initial clinical implementations and promising real-time applications [6,7,16]	Disagreement on readiness: many reviews stress the infancy of clinical adoption, citing infrastructure limitations, real-time processing constraints, and user acceptance barriers [10,11,35]. Also, some highlight hardware limitations in devices like HoloLens [24]	Divergence due to varying surgical environments, technological readiness, and differences in AI system design (standalone vs. server-based). Clinical workflow disruption and surgeon trust issues also contribute
Ethical and regulatory compliance	Most papers acknowledge important ethical concerns, such as patient data privacy, algorithm transparency, and the need for regulatory frameworks. Implementation requires addressing these issues for safe AI adoption [5,10,12,14]	Some studies provide more detailed ethical frameworks or call for standardized guidelines, while others focus mainly on technical performance without extensive ethical discussion [11,18,28]	Differences reflect the scope of reviews (technical vs. comprehensive), geographic regulatory environments, and the maturity of AI applications in clinical contexts. Ethical considerations are evolving alongside technology development
Impact on surgical outcomes	General consensus that AI enhances surgical precision, reduces errors, and improves patient safety, supported by improvements in surgical performance scores and complication prediction [4,19,31]	Some divergence in evidence strength for direct clinical outcome improvements; a few highlight limited prospective clinical trials and lack of long-term outcome data [10,27]	Variability due to predominance of retrospective analyses, limited randomized controlled trials, and early-stage AI tools, mostly validated in simulated or animal models rather than extensive human trials
AI model comparison	Several studies agree that DL techniques (e.g., CNNs) often outperform traditional ML in visual recognition and IOP guidance tasks [6,16,32]. Gradient boosting and random forests are favored for risk prediction	Contrasting perspectives exist on the best algorithms depending on specific tasks; some emphasize interpretability of simpler models over accuracy of complex DL models [12,27]	Differences arise from task-specific requirements, dataset characteristics, need for interpretability vs. accuracy, and computational resource availability. Different surgical applications demand tailored AI approaches
Data quality and availability	Strong agreement that high-quality, large, and standardized datasets are critical for model training, with data limitations being a major bottleneck [5,10-12]	Some studies differ on the sufficiency of current datasets; while some report large multicenter datasets, others highlight scarcity and lack of annotated data as barriers [27,29]	Variances depend on surgical specialty data-sharing cultures, regulatory constraints on patient data, and the availability of annotated surgical videos or imaging. The nascent stage of data infrastructure contributes to disparities

Theoretical Implications

Overall, the findings suggest that AI, specifically ML and DL, is increasingly accurate and reliable in IOP decision-making. This confirms already formulated hypotheses that AI will be able to assist with human cognitive tasks during complex surgeries, providing high-quality real-time guidance and predicting risk [4,5,20]. The literature reviewed demonstrates the importance of integrating data and state-of-the-art algorithms, such as convolutional neural networks and generative adversarial networks, to capture surgical workflow and predict subsequent surgical stages. In this way, context-aware intelligent systems and surgical workflow analysis can be achieved [17,33]. Both the transparency and interpretability of clinical decision-making are called into question by ethical considerations and by the fact that some AI models are black boxes. This will require theoretical clarification of explainability and trust-building in AI systems used intraoperatively [10,12,14]. The lack of autonomy in existing AI applications in robotic surgery, mainly of the assistance or task-autonomy type, suggests that the theories of human-robot collaboration and the gradual adoption of autonomy hold. The shift to AI-specific measures to process outcomes with greater autonomy is consistent with developmental models of surgical AI implementation. The supposition that AI will uncover nonlinear, complex interactions in clinical data that conventional statistical algorithms can overlook is supported by the statistically proven superiority of the AI model over these algorithms in forecasting surgical complications and outcomes [8,28]. The emergence of generative AI as a potential source of real-time feedback and for synthesizing IOP data provides novel theoretical frameworks for AI as an interactive co-worker in the operating room, rather than a passive decision-support system.

Practical Implications

IOP decision support systems based on AI can boost precision, reduce IOP errors, and improve patient safety, potentially leading to mass adoption of the technology by primary surgical specialties as soon as the issues associated with data quality and clinical validation are resolved [5,6]. Even though the application of AI in robotic surgical operations is still at its initial phase and it is not based on high autonomy levels, the potential robotic surgical operation changes it provides, such as advanced metrics, fully automated task performance, and improved surgical training, should be invested in infrastructure and training [18]. The use of AI in the operating room can be integrated as a standard tool to reduce risk and facilitate communication with colleagues through real-time applications such as bleeding detection and hazard identification using computer vision and augmented reality (AR) [23,24]. The lack of external validation, the extremely small sample size, and the improper reporting of AI model performance across multiple populations also underscore the need to implement a standardized assessment framework and regulatory rules to support the deployment of AI in the clinical setting [10,27]. As the verification confirms, the programs are operational for AI training for surgeons, providing an opportunity to develop high-quality surgical skills and ensure adherence to safety standards. That is why the identified programs can be introduced into the surgical training practice session [31]. The innovations in risk stratification in the perioperative setting and the ability of AI models to predict the time spent on each surgery case suggest that they can be used in practice to better organize the operating room workflow, allocate resources, and direct specific cases [26,30].

Current Landscape of AI in Surgical Care

AI and ML applications in surgery span various domains, including preoperative assessment, IOP guidance, and postoperative monitoring [9]. Predictive analytics can assess patient risk factors and optimize surgical planning, while AI-driven imaging technologies, such as AR and computer vision, enhance surgical precision [36]. Robotics-assisted surgery, though still in its infancy in LMICs, holds promise for improving surgical accuracy and accessibility [26]. Moreover, AI-powered telemedicine and remote surgical mentoring can address the shortage of specialized surgeons in rural and underserved areas [37].

AR is another rapidly emerging technology that enhances IOP visualization, allowing surgeons to overlay digital images onto real-time surgical fields [38]. AI-integrated AR can provide real-time guidance, reducing IOP complications and improving surgical efficiency [39]. Such technologies have already been widely adopted in high-income countries and have the potential to be scaled for use in LMICs with appropriate investment and policy support.

Challenges to AI Adoption in LMICs

Despite the promise of AI in surgery, LMICs face various challenges to its adoption. The unavailability of high-speed internet, cloud computing, and AI-compatible surgical equipment in many LMICs is a significant issue [40]. Without these core tools, a surgical AI application is challenging. Another obstacle is data gaps and the need for local context adaptation. In LMICs, the lack of locally relevant surgical data restricts the accuracy and applicability of AI models [41].

Lack of AI competence is another issue. The lack of AI-literate healthcare workers delays AI-driven surgical interventions and technological integration [42]. Since AI applications require significant technological and training investments, financial and policy restrictions are also necessary. Without regulatory frameworks for AI in healthcare, integrating AI into surgical practice is difficult, thereby increasing ambiguity about AI-based solutions. AI-driven surgical treatment may worsen health disparities if not adequately regulated, raising ethical and equality concerns. Without strategic planning, AI developments may benefit urban centers while disregarding rural and underprivileged communities, exacerbating the LMICs' healthcare divide [39].

Limitations

Even with strong performance metrics, the generalizability and robustness of AI models are limited by data quality, heterogeneity, and weaknesses in standardization. The relative lack of large, multi-institutional, and well-annotated IOP datasets is a severe constraint on the ability to design models that can be successfully applied across different clinical sites. Given that the issue of ethics, e.g., patient privacy, algorithm transparency, and AI results impartiality, is not a novel one, they demonstrate the importance of having extensive regulatory interventions at their disposal, as well as interpretable AI software, which will become a key metric in the establishment of trust among clinicians and will go a long way toward comforting patients. The lack of standardized reporting on demographic equity and external validation further complicates the clinical adoption of AI.

Future of AI and ML in Surgical Care for LMICs

AI-driven surgery in LMICs has a bright future if smart investments and collaboration overcome constraints. We advocate training healthcare workers in AI and ML through interdisciplinary collaborations with academic institutions and technological enterprises. Encourage government, corporate sector, and international partnerships to finance AI-based surgical efforts. Designing local AI solutions using LMIC-specific data to increase diagnosis accuracy and relevance. Increasing digital health infrastructure to support AI-powered surgical tools and remote surgery. Policy and ethics to create ethical, egalitarian, and sustainable frameworks for surgical AI adoption. To develop affordable AI-assisted surgical instruments that fit LMIC healthcare budgets and skills.

## Conclusions

This narrative literature review found that AI models' overall performance in IOP decision-making is substantial, with five of six studies reporting AUC values of 0.85-0.95, indicating strong discriminatory power. Moreover, the accuracy performance metric among 22 studies showed high predictability, with accuracy ranging from 80% to 99%, except for one study below 70%, emphasizing the practical feasibility of AI in IOP decision-making. Hence, AI's role in IOP is promising, assisting surgeons' decision-making in the operating room. Hence, ML and DL are highly precise in anatomic detection, surgical phase detection, complication prediction, and real-time event detection. Developments in deep learning architectures, such as convolutional neural networks and generative adversarial networks, have enabled more accurate surgical guidance and the prediction of IOP events, thereby increasing surgical accuracy and potentially reducing errors. However, the model's performance needs to be validated through long-term computational and real-time clinical study designs, ensuring appropriate strategies for data validation and model performance assessment. The narrative review study design focused solely on the narrative synthesis, rather than on data validation (internal or external) or quality assessment of the included studies. Future researchers are encouraged to perform systematic reviews to validate the evidence. Although AI tools are still in the early stages of clinical integration, they offer potential, especially in robotic surgery, IOP guidance, and perioperative risk assessment. Technical demonstrations of real-time AI applications have occurred, and initial surgeon feedback has been overwhelmingly positive, particularly for optimizing workflows and surgical education through AI-assisted coaching programs. Nevertheless, there are infrastructural, regulatory, and cultural barriers to adoption in low-resource environments, such as the need to support large computational resources, provide surgeon training, and modify existing workflows.
